# From Awareness to Action: Addressing Folic Acid Supplementation in Western India Among Women of Reproductive Age

**DOI:** 10.7759/cureus.70173

**Published:** 2024-09-25

**Authors:** Subhodip Mitra, Rashmi Ranjan Guru, Sammita Jadhav, Usman U Saurayi, Rahul Kumar

**Affiliations:** 1 Hospital Administration, All India Institute of Medical Sciences, Kalyani, Kalyani, IND; 2 Hospital Administration, Symbiosis Institute of Health Sciences, Pune, IND; 3 Hospital Administration, Postgraduate Institute of Medical Education and Research, Chandigarh, IND; 4 Health Sciences, Symbiosis Institute of Health Sciences, Pune, IND; 5 Hospital and Healthcare Administration, Symbiosis Institute of Health Sciences, Pune, IND

**Keywords:** folic acid supplementation, health education & awareness, iron-deficiency, neural tube defects (ntds), preconception health

## Abstract

Background

Folate, a vitamin B9, can be sourced naturally in the diet or the form of supplements. Studies highlight the prevention of neural tube abnormalities in women of reproductive age. To prevent these, low daily doses of FA (400-800 μg) are recommended for all women planning pregnancy, with higher doses for those with previous NTD-affected pregnancies. Folic acid supplementation lowers the risk of NTDs, other birth defects, and obstetrical complications.

Methods

The study explored awareness of and knowledge of folic acid supplements among women of childbearing age in Pune City, western Maharashtra, India. The cross-sectional survey was carried out at Symbiosis University Hospital and Research Centre (SUHRC), involving 300 female participants aged 16-44 years. The study utilized a structured questionnaire to evaluate participants’ knowledge of folic acid supplements, their benefits, and usage patterns.

Results

Use as well as awareness of folic acid supplements was strongly associated with educational attainment. The awareness was low or none in participants who never attended school, and highest among the university graduates. The study included 300 women aged 16-45. About 43% of the study participants were between the ages of 23 and 29 years old; 57.7% were single, and 59.3% among them were university graduates. 59.7% of the study participants knew about folic acid supplements, but only 20% took them regularly. Knowledge about ideal timing and benefits was limited among them. 38% correctly identified prepregnancy as the ideal time to start. Only 18% knew it prevents neural tube defects, and 27% knew it could be obtained naturally.

Conclusion

The study highlights a lack of detailed knowledge about folic acid supplements among the study participants. Recommendations to enhance the supplement’s intake include public health campaigns, enhanced healthcare provider education, easy-to-read informational materials, and strengthening government supplement programs to improve awareness and food fortification. Further research on consumption barriers for the supplement needs to be carried out.

## Introduction

Several studies have suggested a strong need for better health education and the participation of medical professionals in educating women about the importance of folic acid supplementation, particularly during the preconception stage. Promoting FA supplementation prior to pregnancy is essential for any community, particularly in those with a moderate level of education, nations with low incomes, and those with established histories of neural tube abnormalities (NTDs), diabetes, obesity, and epilepsy [1,2].

Folate, a water-soluble group of vitamin B9, is found in dietary supplements as folic acid, which is a chemically manufactured type of vitamin. It is present naturally only in minimal quantities in food; for these reasons, foods enriched with FA and dietary supplements containing FA are the primary sources of dietary folate [3].

Conversely, among the most frequent causes of birth defects involving the central nervous system are neural tube malformations. Neural tube closure typically starts around day 22 of gestation and is finished in 4-6 days. This brief window of opportunity is a period of marked vulnerability during which anomalies that result in both major and minor birth malformations may begin. Depending on the severity of the anomaly, any level of the neural tube may be impacted, potentially leading to meningomyeloceles or encephaloceles, which are cystic dilatation of the spinal cord or brain coverings. Anencephaly, being the most severe neural tube defect, occurs as a result of a fault in the closure of the rostral neural tube, which prevents the brain from developing [4].

Regarding all females who are planning for pregnancy, a small amount of folic acid each day is highly advised prior to pregnancy, as well as during the length of the pregnancy and breastfeeding [5]. High-dose folic acid supplements should only be taken by women who desire to become pregnant and whose previous pregnancy was affected by a neural tube defect or another folate-sensitive congenital disease. Taking dietary folate or oral folic acid supplements reduces the risk of obstetrical problems, neural tube defects, and other folate-sensitive birth disorders [5]. According to a study [6], a daily supplement providing 400-800 μg (0.4-0.8 mg) of folic acid is advised for all women who are capable of becoming pregnant or who are considering a pregnancy. The risk of NTD has been found to be higher in women who have been exposed to pharmaceuticals that have an anti-folic acid activity; these medications include methotrexate, sulfonamides, and antiepileptic drugs (carbamazepine, valproate, and barbiturates) [5]. Folate has widely been examined to have a crucial role in markedly decreasing the possibility of neural tube defects (NTDs) in fetuses as well as decreasing the risk of megaloblastic anemia in women of reproductive age [7].

Rationale

Neural tube defects (NTDs) are the most prevalent birth condition in India. About 7% of deaths among children under the age of five in India are attributed to birth abnormalities. In comparison to other parts of the world, India has a high prevalence of neural tube defects, according to data from the World Health Organization (2013). In India, the random effect or pooled birth prevalence of neural tube abnormalities is 4.5 per 1000 live births (95% CI 4.2 to 4.9) [8]. In India, most parents learn about their deformed child only after it is born due to unplanned pregnancies, a lack of knowledge about the advantages of periconceptional folate supplementation, and the lack of some specific prenatal diagnostic testing [8]. According to a study conducted in India, the majority of research participants knew very little about taking supplements containing folic acid. Also, they suggested that health education as well as information dissemination regarding folic acid supplements ought to be put into practice in order to raise community women's awareness regarding folic acid supplementation. Further, improved knowledge of folic acid supplementation can encourage folic acid intake and may shield newborns from NTDs [9].

## Materials and methods

The cross-sectional survey was conducted at Symbiosis University Hospital and Research Centre, a charitable medical center located in Pune city, in the western part of India.

The inclusion criteria included female gender within the reproductive age group, willingness to participate in the study, and being a resident of Pune City. Individuals who were critically ill and couldn’t communicate were not included in the study. The survey was conducted between March 2024 and June 2024 and consisted of 300 participants who were randomly selected and included in the study following informed verbal consent. Among them were female students from different institutes studying at the university, stable female patients presenting to the hospital’s outpatient department for medical care, ranging from mild to moderate medical problems, inpatients in different wards of the hospital, and female staff working in and outside of the hospital but within the university. The participants were from different socioeconomic backgrounds, different levels of education, and different age groups, ranging from 16 to 44 years. A structured questionnaire using a Likert scale was developed in English, consisting of close-ended questions. It was then translated into Marathi by an expert who fluently speaks both English and the local language that is commonly used in the city (Marathi). The translated questionnaire was given to other experts for thorough review and verification before being dispatched to the participants; it was also distributed as self-administered, electronically in the form of a Google form to individuals who were digitally literate, and also in printed form as a face-to-face interview conducted by trained nurses to individuals who were not digitally competent and illiterate. The filled-out questionnaires were received both digitally and non-digitally. The questionnaire constituted of two parts; the initial section focused on social and demographic data of the participants (educational level, marital status, employment status, and age) as well as parity. The second section was to explore the participants’ knowledge regarding folic acid and its sources. Also, a question concerning the ideal time to take folic acid in order to prevent some birth defects, specifically NTD, was assessed; we also conducted assessments on the natural source of folic acid, attitudes toward folic acid consumption, and the frequency of folic acid supplementation.

Statistical analysis

IBM Corp. Released 2011. IBM SPSS Statistics for Windows, Version 20.0. Armonk, NY: IBM Corp. was utilized for the statistical analysis of this study. The mean, mode, and standard deviation, as well as the standard error of the mean, were computed for the demographic characteristics. The frequencies, percentages, valid percentages, and cumulative percentages of all the variables were analyzed.

Ethical consideration

The Symbiosis International University institutional ethical committee approved the study with reference number SIU/IEC/434. Every participant gave their informed consent before consenting to the study. Autonomy and rights to personal opinions and decisions were upheld for each of the participants, as their participation was voluntary. Complete confidentiality was maintained for all information gathered from the respondents.

## Results

As mentioned above, all 300 participants were female of reproductive age, ranging from 16 to 45 years of age. Among these, the highest were the participants within the age range of 16-22 (10%), 23-29 (43%), followed by 30-36 (38.7%), 37-43 (6.7%), and 44-45 years of age being the least (1.7%). Most of the participants were not married (57.7%), and also from different socio-demographic backgrounds as depicted in Table 1. Among the participants were women who had given birth either once or multiple times before (43.6%). The majority of the participants, about 59.3%, attended university, followed by secondary school (31.0%), and a handful of the participants had only primary school as their highest level of educational qualification (5.3%). Again, most of the respondents were unemployed at the time of the study (69.7%), with just 30.3% employed. 59.7% of the study participants knew about folic acid supplements, among whom were mostly either university graduates or were currently studying at the university at the time of the study. The main source of information for the supplements was from healthcare personnel. A minimal portion of the university students (0.58%) never heard anything about folic acid supplements or used them before. 40.3% of the study participants had never heard of folic acid supplements, among whom were mostly uneducated.

**Table 1:** Shows the demographic data of the respondents Total no. of respondents (N)=300.

Demographic Attributes	Frequency	Percentage
Age		
16-22	30	10%
23-29	129	43%
30-36	116	38.7%
37-43	20	6.7%
44+	5	1.7%
Marital Status		
Single	173	57.7%
Married	127	42.3%
Educational level		
None	13	4.3%
Primary School	16	5.3%
Secondary School	93	31%
University	178	59.3%
Parity		
Nulliparous	169	56.3%
1-3	131	43.7%
4+	0	0.0%
Employment Status		
Employed	209	69.7%
Not employed	91	30.3%

Of the 59.7% of the participants who were aware of the supplements, only 20% were taking them regularly; the remaining were either not taking them or were taking them but not regularly.

Also, knowledge regarding the ideal period of time to take folic acid supplements was assessed. 38% of the respondents who were aware of the supplements mentioned that the ideal period to take them should be right before planning for pregnancy; 20% of them chose the period after becoming pregnant; 1.7% chose the time period after giving birth as the ideal period to start taking the supplements, while 40.3% were not sure of the ideal time to take the supplement. Among the participants who gave birth before, 46.83% took the supplements during their pregnancy, while 53.17% were not sure whether they were given the supplements or not. Among those who had never had a pregnancy before, 68.57% believe that a folic acid supplement is important to them, while 31.43% are not sure of the supplement’s status. Among the participants who heard of the supplement, not all of them were aware of the supplement’s benefits and that it can prevent birth defects; 18% of them knew that it could prevent NTD when taken earlier before conception, while 82% didn’t know that it can prevent NTD. Of the 178 participants who were aware of the supplement, 73% either didn’t know or were not sure that it could be obtained naturally; just 27% were aware that the supplement could be naturally obtained, as shown in Table 2.

**Table 2:** Distribution of responses based on the knowledge of FA supplements among women of reproductive age Total no. of respondents (n)=300

Questions		Responses, n(%)				
		Strongly agree	Agree	Not sure	Disagree	Strongly disagree
1	I don’t know about folic acid supplement.	24(8%)	97(32.3%)	0(0%)	149(49.7%)	30(10%)
2	I know about folic acid supplement.	41(13.7%)	138(46%)	0(0%)	102(34%)	19(6.3%)
3	I know about folic acid supplement, and I am currently taking it regularly.	2(0.7%)	39(13%)	0(%)	246(82%)	13(4.3%)
4	I know about folic acid supplement, but I am not currently taking it regularly.	6(2%)	60(20%)	0(%)	229(76.3%)	5(1.7%)
5	I know about folic acid supplement, but I am not taking it.	12(4%)	75(25%)	0(0%)	207(69%)	6(2%)
6	Folic acid supplement should be taken right before I get pregnant.	6(2%)	108(36%)	159(53%)	24(8%)	3(1%)
7	Folic acid supplement should be taken after I get pregnant.	4](1.3%)	56(18.7%)	146(48.7%)	88(29.3%)	6(2%)
8	Folic acid supplement should only be taken after giving birth.	0(0%)	5(1.7%)	151(50.3%)	127(42.3%)	17(5.7%)
9	Folic acid supplements should be taken once daily.	7(2.3%)	163(54.3%)	127(42.3%)	3(1%)	0(0%)
10	I know the benefits of folic acid supplement.	1(0.3%)	53(17.7%)	75(25%)	171(57%)	0(0%)
11	Folic acid can also be obtained naturally.	6(2%)	73(24.3%)	211(70.3%)	10(3.3%)	0(0%)
12	Folic acid supplements are expensive.	0(0%)	2(0.7%)	129(43%)	162(54%)	7(2.3%)
13	Folic acid supplement is injurious to the body.	0(0%)	2(0.7%)	125(41.7%)	158(52.7%)	15(5%)
14	I have been pregnant before, and I didn’t take folic acid supplement.	0(0%)	0(0%)	67(22.3%)	59(19.7%)	174(58%)
15	I have been pregnant before, and I took folic acid supplement during the pregnancy.	4(1.3%)	55(18.3%)	67(22.3%)	6(2.0%)	168(56%)
16	I have never been pregnant before; therefore, folic acid supplement is not important to me.	0(0%)	0(0%)	55(18.3%)	112(37.3%)	133(44.3%)
17	I have never been pregnant, but folic acid supplement is still important to me.	10(3.3%)	110(36.6%)	55(18.3%)	0(0%)	125(41.6%)

Educational level was strongly associated with both folic acid supplement use and awareness. The awareness was low or none in participants who never attended school, and it was highest among participants who attended school.

## Discussion

The global estimates of the prevalence of NTD (range: 0.3-199.4 per 10,000 births) are 66.2 out of every 10,000 births in India [10]. The results of this study demonstrated a strong relationship between an individual's educational attainment and their awareness of folic acid supplements. The educated participants, specifically those undergoing a college degree or having acquired one college, had more knowledge about the supplements, whereas poor knowledge of the supplements was seen among the uneducated participants. The findings were similar to research conducted [11] in the UAE; in a study [12] in Japan, as well as Panasiuk and Hozyasz (2013) in Poland, the proportion of Polish women who reported taking folic acid throughout their first trimester and before getting pregnant was higher in those who had completed college or university (75% versus 71.5% for preparation). Despite the availability and low price of the supplements, the awareness and knowledge of the supplements were not encouraging among the study participants, specifically among the uneducated respondents. These were evidenced by the poor value obtained among the overall study participants who were aware of the supplements (59.7%). This study was in line with a study conducted in the UAE, Turkey, and Thailand, in which they found out that in the UAE, 46.4% acknowledged having heard of folic acid supplements [11], 42.2% in Turkey [13], and as low as 24.4% among Thai women in a study [14]. However, a study [15] conducted among medical students in Ukraine, [16] Saudi Arabia, and Thai women [14] revealed the opposite: 96.5% of the participants were aware of the supplements among the medical students in Ukraine, 93.1% in Tabuk, KSA, and 90% in Taipei. A study in India found that as many as 77.9% of the respondents possessed inadequate knowledge of FA supplements [9]. Of the 178 who were aware of the supplement, 20% of them reported taking the supplements regularly, and 80% admitted either not taking the supplement regularly or not taking it at all. This was contrary to a study again conducted in the UAE [11], which revealed that 44.5% of the participants who were aware of the supplements were actually taking them regularly, but a study conducted in Ukraine among medical students [15] reported that just 10% of the participants were consuming synthetic FA regularly, 9.7% from a study conducted in Thailand [14], 15.6% from research done by authors from Thailand [14], and as low as 3.5% from a study conducted in Japan [12]. However, the value obtained in our study was similar to the value obtained from a study conducted in Glasgow [17], which revealed only 21% of respondents among those who were aware of the supplements were actually taking them regularly. The discrepancy between the use and awareness of the supplements could be a result of most pregnancies being unplanned or that expectant mothers are not given adequate information regarding the supplement’s benefits. Also, in our study, among the participants who were aware of the supplements, 63.69% mentioned that the supplements should be taken before getting pregnant, 33.52% believed that they should be taken following pregnancy, and 2.79% believed that they should be taken after giving birth. The above was not in line with a study conducted in Scotland, in which they found out that 66% of the participants who were aware of folic acid supplements knew that the supplements should be taken when trying to conceive, and 34% of them mentioned that they should be taken during the first trimester [17]. However, a study again conducted in India [9] revealed the opposite; they discovered that just 1.3% of the women were aware that taking folic acid supplements prior to becoming pregnant is necessary, and 28.8% of the women were aware that taking the supplements during the early stage of pregnancy is also important. Again, in our study, just 26.3% of the participants who heard about the supplements knew that they could be obtained naturally. This was not similar to research carried out among medical students in Ukraine [15], in which they discovered that 95.6% of the respondents knew that supplements could be obtained from their diet. Among the participants who were aware of the supplement, not all of them were aware of the supplement’s specific benefits, such as its role in preventing abnormalities in the neural tube. Among the 178 individuals who heard of the supplement, only 18% were aware that it could prevent birth abnormalities, particularly neural tube abnormalities. This study was almost identical to one [18] in KSA [19]. Among Qatari women, in a European study [20], they also found low knowledge regarding the specific benefit of the supplement among the participants (that it can prevent NTD): 12%, 14%, and 17%, respectively. The main source of the supplement’s information among those who were aware was mainly healthcare professionals; it was comparable to a study conducted in the UAE [11], and nearly similar to a study conducted in Japan [12].

In accordance with the reproductive, maternity, newborn, child, and adolescent (RMNCH+A) strategy, accredited social health activists (ASHAs) are in charge of raising community knowledge about folic acid supplementation [21]. Activities that can increase women of childbearing age's awareness and knowledge of supplements that contain folic acid, especially among the uneducated communities, regardless of the women's intention to become pregnant, should be mobilized extensively in order to achieve full awareness of the supplement, given the low knowledge of the supplement observed in our study participants. These can be disseminated through various channels. To improve the iron and folate status of women of reproductive age, the Indian government has implemented a weekly supplement distribution program that includes tablets with a combination of 100 mg elemental iron and 500μg folic acid. However, in reality, all of the young ladies and adolescents that were encountered admitted that they didn't always take these pills as prescribed [22]. This could be a result of the supplement’s lack of proper knowledge. Relevant health information regarding the obvious benefits of the supplement, the frequency of its intake, and the best time to begin taking should be shared, especially among those who are aware of the supplements. Similarly, it would also be a good idea to hand out information explaining the benefits of folic acid to patients visiting medical institutions in both English and the local language.

A range of strategies can be collated from different studies and used to create public health campaigns to raise knowledge of folic acid's ability to protect against NTDs. It is important that these campaigns target women who desire to be pregnant. Discussing the value of folic acid in avoiding neural tube abnormalities (NTDs) with medical practitioners should be a priority for all women who are of reproductive age. India does not yet have a fortification policy in place [22]. However, one practical method for supplying a sufficient amount of folate is food fortification. The number of elective fetal terminations for neural tube defects (NTDs) could significantly decline as a result of the fortification policy. In a Canadian study, the risk of neural tube abnormalities was considerably lower in diets supplemented with folic acid [23]. Due to the fact that ''participants' inadequate intake of folic acid supplements, despite their prior knowledge of the supplements'', more research is necessary to determine the factors that motivate people to take a daily multivitamin that includes folic acid.

Limitations

One limitation of our study is that it was conducted exclusively at the university hospital. The participants included women who were visiting the hospital for medical reasons as well as students who were explicitly studying at the institution. Furthermore, some demographic data that may not have been included in the research, as well as the smaller sample size used in the investigation, may prevent the research from being generalized to the entire community.

Recommendations

The study recommends a smorgasbord of efforts that need to be initiated by healthcare administrators and public health professionals in close collaboration with the government. Implementation of a comprehensive public health campaign to raise awareness regarding the preventive role of folic acid against neural tube defects (NTD) can be one of those efforts. Empowering healthcare providers to educate women about folic acid supplementation during preconception and early pregnancy can also be a step toward spreading awareness of folic acid among the targeted population. Efforts such as the development and distribution of easy-to-read informational materials in both English and local languages. Collaboration with the government in making newer and innovative food fortification policies to increase folate intake across all socioeconomic groups while at the same time strengthening the existing government programs for supplement distribution and improving compliance through better education. The study also recommends further research to understand barriers to regular folic acid consumption among aware individuals.

By implementing these measures, the authors are confident that it will be feasible to increase the awareness and consumption of folic acid among women of reproductive age, possibly lowering the frequency of folate-sensitive congenital abnormalities in the area, such as neural tube defects. Continued efforts in research, education, and policy implementation are crucial to addressing this important public health issue.

## Conclusions

The study reveals a significant gap regarding the awareness as well as the knowledge of folic acid supplements among women of reproductive age in Pune City. While above half of the participants were aware of FA supplements, a detailed understanding of their benefits and proper usage was lacking. The strong correlation between education level and supplement awareness underscores the need for targeted educational initiatives, especially for women with lower educational backgrounds.

## References

[1] Tsegai MB, Berhe AH, Tesfaezgi SB, Weldemariam DG, Petros KT, Weldetinsae HB, Tesfamariam EH (2023). Knowledge, attitude, and practice regarding supplemental iron and folic acid amongst women delivering in Edaga-Hamus community hospital: a cross-sectional study in Asmara, Eritrea. Int J Womens Health.

[2] Medawar G, Wehbe T, Jaoude EA (2019). Awareness and use of folic acid among women of childbearing age. Ann Glob Health.

[3] Ferrazzi E, Tiso G, Di Martino D (2020). Folic acid versus 5- methyl tetrahydrofolate supplementation in pregnancy. Eur J Obstet Gynecol Reprod Biol.

[4] King T (2007). Nervous system. Els Integ Path.

[5] Matok I, Gorodischer R, Koren G, Landau D, Wiznitzer A, Levy A (2009). Exposure to folic acid antagonists during the first trimester of pregnancy and the risk of major malformations. Br J Clin Pharmacol.

[6] (2009). Folic acid for the prevention of neural tube defects: U.S. Preventive Services Task Force recommendation statement. Ann Intern Med.

[7] Zheng Y, Cantley LC (2019). Toward a better understanding of folate metabolism in health and disease. J Exp Med.

[8] Rai SK, Singh R, Pandey S (2016). High incidence of neural tube defects in Northern part of India. Asian J Neurosurg.

[9] Pal A, Shukla AK, Santra A (2023). Awareness about folic acid supplementation in first-trimester pregnant women of rural Raipur District, Chhattisgarh, and its determinants: a cross-sectional study. Cureus.

[10] Zaganjor I, Sekkarie A, Tsang BL (2016). Describing the prevalence of neural tube defects worldwide: a systematic literature review. PLoS One.

[11] Abdulrazzaq Y, Al-Gazali L, Bener A (2003). Folic acid awareness and intake survey in the United Arab Emirates. Reproductive toxicology.

[12] Kondo A, Yamamoto S, Inoue H, Watanabe J, Tada K, Yoshimoto N (2009). Folic acid in the prevention of neural tube defects: awareness among laywomen and healthcare providers in Japan. Congenit Anom (Kyoto).

[13] Köken GN, Derbent AU, Erol O, Saygın N, Ayık H, Karaca M (2013). Awareness and use of folic acid among reproductive age and pregnant women. J Turk Ger Gynecol Assoc.

[14] Nawapun K, Phupong V (2007). Awareness of the benefits of folic acid and prevalence of the use of folic acid supplements to prevent neural tube defects among Thai women. Arch Gynecol Obstet.

[15] Hlushko K, Boyarchuk O, Kinash M (2021). Awareness of folic acid use and its effects among medical students in Ukraine. Wia Lek Med Adv.

[16] Alblowi S, Alomayri M (2018). Assessment of knowledge, awareness, and behavior of folic acid use among females during the childbearing period in Tabuk City. Egy Jr Hos Med.

[17] McGovern E, Moss H, Grewal G, Taylor A, Bjornsson S, Pell J (1997). Factors affecting the use of folic acid supplements in pregnant women in Glasgow. Br J Gen Pract.

[18] Kari JA, Bardisi ES, Baitalmal RM, Ageely GA (2008). Folic acid awareness among female college students: neural tube defects prevention. Saudi Med J.

[19] Bener A, Al Maadid MG, Al-Bast DA, Al-Marri S (2006). Maternal knowledge, attitude and practice on folic acid intake among Arabian Qatari women. Reprod Toxicol.

[20] Bitzer J, von Stenglin A, Bannemerschult R (2013). Women's awareness and periconceptional use of folic acid: data from a large European survey. Int J Womens Health.

[21] Ministry of Health & Family Welfare Government of India. (2013 A strategic approach to reproductive, maternal, newborn, child and adolescent health (RMNCH+A) in India. https://nhm.gov.in/images/pdf/RMNCH.

[22] Vora RM, Antony AC (2022). The unresolved tragedy of neural-tube defects in India: the case for folate- and vitamin-B(12)-fortified tea for prevention. J Indian Assoc Pediatr Surg.

[23] De Wals P, Tairou F, Van Allen MI (2007). Reduction in neural-tube defects after folic acid fortification in Canada. N Engl J Med.

